# Validation of Model-Based Melt Viscosity in Hot-Melt Extrusion Numerical Simulation

**DOI:** 10.3390/pharmaceutics10030132

**Published:** 2018-08-18

**Authors:** Esther S. Bochmann, Andreas Gryczke, Karl G. Wagner

**Affiliations:** 1 Department of Pharmaceutical Technology and Biopharmaceutics, University of Bonn, 53121 Bonn, Germany; esther.bochmann@uni-bonn.de; 2 AbbVie Deutschland GmbH & Co. KG, 67061 Ludwigshafen am Rhein, Germany; andreas.gryczke@abbvie.com

**Keywords:** hot-melt extrusion, melt rheology, glass transition temperature, amorphous solid dispersion, simulation, prediction model

## Abstract

A validation for the use of model-based melt viscosity in hot-melt extrusion numerical simulations was presented. Here, the melt viscosity of an amorphous solid dispersion (ASD) was calculated by using its glass transition temperature (*T_g_*) and the rheological flow profile of the pure polymeric matrix. All further required physical properties were taken from the pure polymer. For forming the ASDs, four active pharmaceutical ingredients (APIs), that had not been considered in first place to establish the correlation between *T_g_* and melt viscosity were examined. The ASDs were characterized in terms of density, specific heat capacity, melt rheology, API solubility in the polymeric matrix, and deviation from the Couchman–Karasz fit to, identify the influencing factors of the accuracy of the simulation using model-based melt viscosity. Furthermore, the energy consumption of the hot-melt extrusion (HME) experiments, conventional simulation, and simulation using model-based melt viscosity were compared. It was shown, with few exceptions, that the use of model-based melt viscosity in terms of the HME simulation did not reduce the accuracy of the computation outcome. The commercial one-dimensional (1D) simulation software Ludovic^®^ was used to conduct all of the numerical computation. As model excipients, vinylpyrrolidone-vinyl acetate copolymer (COP) in combination with four APIs (celecoxib, loratadine, naproxen, and praziquantel) were investigated to form the ASDs.

## 1. Introduction

Nowadays, one of the major challenges in pharmaceutical small molecule formulation development is the increasing number of poorly soluble active pharmaceutical ingredients (APIs) and the respective poor bioavailability. To enhance the solubility of these APIs, the formation and stabilization of amorphous solid dispersions (ASDs) is commonly used. For manufacturing ASDs, hot-melt extrusion (HME), as a continuous and solvent-free process, was often reported [1,2,3,4,5,6]. Unfortunately, HME is a time and API-consuming procedure, especially in early ASD formulation development, as the various process parameters, such as screw speed, throughput, screw configuration, and temperature profile lead to a complex multivariable process, which is challenging to optimize or scale-up [7,8,9].

Simulation has been found to be a valid and useful tool to ease HME optimization, scale-up, and to improve the comprehension of the processes itself in order to reduce the remaining API crystallinity and degradation [7,9,10,11,12]. By using simulation, the temperature, pressure, and shear profiles along the screws are computed and the process window for HME can be defined [13]. However, the main drawback of HME simulation is the need for experimental product input data to conduct computation. In some cases, these data are not easy to access, especially in terms of melt rheology and thermosensitive APIs. Furthermore, the use of simulation for early formulation screening is limited, as the physicochemical characteristics would need to be laboriously measured for every formulation under consideration. As simulation is an inevitable tool to evaluate a potential adiabatic scale-up from small- to large-scale extruders, the above-mentioned drawbacks of HME simulation need to be solved [9].

Several challenges in performing the HME simulation were already addressed by several researchers. In the case of screw configuration optimization, different algorithms and process modellings have been used to define the best operating conditions for an extrusion process [12,14]. Investigations into improve the understanding of the extruder performance in mixing capability, mixing elements, and kneading blocks as a function of the staggering angle were evaluated [15]. It was shown that distributive mixing is more related to the staggering angle than to the disc width, and that mixing elements did not performed significantly better than normal conveying elements. Furthermore, the pressure-dependent wall slippage at the barrel and screw surface as well as the modelling fillers in the HME process have been investigated, which is especially important for the extrusion simulation with melt suspensions [16,17]. Investigations on the simulation of residence time distributions (RTD) revealed that the specific throughput, a ratio of throughput over screw speed, is one of the key process parameters in order to control RTD and to determine the flow conditions during extrusion [18,19].

In our recent work, we proposed a procedure to model the melt viscosity of an ASD by using only its glass transition temperature (*T_g_*) and the rheological flow profile of the pure polymeric matrix [20]. It simplifies the application of the HME simulation by reducing the required physicochemical characterization of the ASD. Therefore, the experimental effort was decreased without compromising the accuracy of the computation, which has been proven by comparing the simulation using model-based melt viscosity, conventional simulation, and the data of experimental trials [21]. However, all of the investigated APIs so far were already used to establish the *T_g_*-melt viscosity correlation in the first place, and thus a validation with new APIs is needed. 

Therefore, the purpose of this work was to ease the HME simulation by providing evidence for the *T_g_*-melt viscosity correlation. Four APIs (celecoxib, loratadine, naproxen, and praziquantel), which were not used to establish the *T_g_*-melt viscosity correlation in the first place, were used to form model ASDs of various *T_g_*. As the applied process simulation is not able to predict the physical stability of the ASDs, this paper focuses on the process simulation only. The physical stability of the ASDs would need to be evaluated by other models, as proposed by various authors [22,23,24,25]. The ASDs were further characterized in terms of density, specific heat capacity, melt rheology, API solubility in the polymeric matrix, and deviation from the Couchman–Karasz fit. The influence of these factors to the accuracy of the simulation using model-based melt viscosity was evaluated, whereas the density, specific heat capacity, and melt rheology are the input parameter for the simulation itself. The commercial one-dimensional (1D) simulation software, Ludovic^®^ (Sciences Computers Consultants, Saint Etienne, France), was used to conduct the conventional HME simulation and simulation by using model-based melt viscosity and further physicochemical characteristics of the pure polymeric matrix only. Both simulation procedures were compared to the extrusion trials by means of energy consumptions.

## 2. Materials and Methods 

### 2.1. Material

Praziquantel (PZQ) was obtained from Divis Laboratories Ltd. (Telangana, India), loratadine (LOR) was purchased from Sris Pharmaceutials (Telangana, India), naproxen (NAP) was received from Sigma Aldrich (St. Louis, MO, USA), and celecoxib (CXB) was obtained from Cadila Pharmaceuticals Ltd. (Ahmedabad, India). Vinylpyrrolidone-vinyl acetate copolymer (copovidone, Kollidon^®^ VA 64, COP) was kindly donated by BASF SE (Ludwigshafen, Germany) (Table 1). All of the investigated APIs belong to BCS (Biopharmaceutics classification system) Class II and were thermally stable over the applied temperature range. None of the selected APIs were used to establish the *T_g_*-melt viscosity correlation in the first place, and thus served for model validation [20,21].

### 2.2. Methods

#### 2.2.1. Helium Pycnometry

The true density of the powder blends and extrudates were measured using the helium pycnometer AccuPyc 1330 (Micromeritics GmbH, Norcross, GA, USA) with 20 purge cycles at a fill pressure of 136.86 kPag. The samples were analyzed in 25 runs or until a standard deviation of 0.01% was reached, using a fill pressure of 136.86 kPag and an equilibration rate of 0.0345 kPag/min. The procedure was repeated two times for every material. The true density of the powder blends and extrudates were used as the input parameters for the HME simulation software, Ludovic^®^.

#### 2.2.2. Differential Scanning Calorimetry (DSC)

In order to identify the heat capacities and glass transition temperatures of the investigated blends, a DSC 2 (Mettler Toledo, Gießen, Germany) was used. It was equipped with an auto sampler, nitrogen cooling, and nitrogen as purge gas (30 mL/min), and the system was calibrated with n-octane, indium and zinc standards. In the case of the *T_g_*-determination as a function of the API weight fraction, at least three samples for each mixture, of approximately 10 mg, were analyzed in 40 μL aluminum pans with a pierced lid. The *T_g_*-determination was conducted after the annealing of the sample in order to define the solubility of the API in the polymeric melt. Please see Section 2.2.3 for a more detailed description of the DSC method.

The heat capacities were measured using a sapphire standard in TOPEM^®^ mode (modulated DSC) with 1 K pulse height, 15–30 s pulse width, and an underlying heating rate of 2 K/min. For every blend, three samples of approximately 10 mg were weightwere put in 40 μL aluminum pans with pierced lids. All of the pans used, including the reference and the pan with the sapphire standard, did not differ more than 0.1 mg in weight from each other. All of the samples were annealed at elevated temperatures for a homogenous API-distribution during the subsequent heat capacity measurement. Every blend for DSC was prepared by using a MM400 ball mill (Retsch GmbH, Haan, Germany) with 30 Hz and 3 × 5 min milling cycles.

#### 2.2.3. Solubility Determination via DSC

To determine the API solubility in vinylpyrrolidone-vinyl acetate copolymer (COP), a protocol of our previous work was used [23]. It determines the solubility of the APIs indirectly, using the glass transition temperature (*T_g_*). The method itself consists of an annealing step and a subsequent *T_g_* analysis of the annealed sample, as well as a further *T_g_* analysis of a completely molten and amorphous sample at elevated temperatures (*T* > *T_m_*).

In more detail, every sample was annealed at a temperature approximately 60 °C above the predicted *T_g, blend_* by the Couchman–Karasz fit, so as to enable a viscous system that promotes an equilibrated state of the solubilized API at the annealing temperature (*T_Annealing_*). Subsequently, the sample is cooled and heated again by 10 K/min to determine the *T_g_* of this annealed sample. The mentioned heating step ended 10 K above the melting point of the API to obtain a completely amorphous system, which is further analyzed by a cooling–heating cycle with 10 K/min to evaluate the *T_g_* of this amorphous system.

This procedure is conducted by using different API/polymer weight fractions. At the end, the first determined *T_g_* serves as an indicator of the soluble API fraction at *T_Annealing_*, while the second *T_g_* was used for the characterization of the weight fraction-dependent curve progression of *T_g_* by employing the Brostow Chiu Kalogeras Vassilikou-Dova fit (BCKV-fit, Equation (1)),

(1) $$\;T_{g}=w_{1}T_{g,1}+\left(1-w_{1}\right)T_{g,2}+w_{1}\left(1-w_{1}\right)\left[a_{0}+a_{1}\left(2w_{1}-1\right)+a_{2}{\left(2w_{1}-1\right)}^{2}\right]\;$$

where *a*_0_, *a*_1_, and *a*_2_ are variables [27]. The polynomial form of the BCKV-fit enables the consideration of positive and negative deviation from the Couchman–Karasz fit (CK-fit, Equations (2) and (3)). It is therefore appropriate for the identification of the solubilized API fraction in the annealed samples, by employing the API weight fraction-dependent curve progression of glass transition temperature.

(2) $$T_{g}=\frac{w_{1}T_{g,1}+k_{CK}\left(1-w_{1}\right)T_{g,2}}{w_{1}+k_{CK}\left(1-w_{1}\right)},\;\mathrm{with}\;\Delta T_{g}=T_{g,2}-T_{g,1}$$

(3) $$\;k_{CK}=\frac{\Delta C_{p,2}}{\Delta C_{p,1}}\;$$

In the Couchman–Karasz equation, *w* is the weight fraction, *k_CK_* is the Couchman–Karasz constant *C_p_* the heat capacity step at *T_g_*, and the sub-scripts 1 and 2 refer to the API and polymer, respectively [28]. 

To predict an API solubility phase diagram, the soluble API fraction at a respective temperature was fitted using Equation (4),

(4) $$\;T_{Annealing}=y_{0}+A\times exp^{R_{0}\times x}\;$$

where *x* is the soluble API fraction at the respective temperature, *A* and *R*_0_ are fitting constants, and *y*_0_ corresponds to the API melting point, but was set as a variable. In general, solubility is referred to as an extrapolation of the dissolved API at 25 °C. Thus, the solubility curve represents the condition at which a crystalline API is solubilized by the polymeric matrix, forming a one phase amorphous solid dispersion.

The obtained solubility curve was confirmed by *x*-ray powder diffraction (XRPD) measurements, which were in good accordance to the DSC findings (data not shown). In most cases, the XRPD results showed a slightly lower solubility of approximately 5% the API in the polymeric melt than in DSC, as XRPD is more sensitive to crystalline residuals than DSC.

#### 2.2.4. Small Amplitude Oscillatory Shear (SAOS) Measurements

The rheometer Haake^®^ MARS^®^ III of Thermo Scientific (Karlsruhe, Germany), equipped with a 20 mm plate–plate geometry, was used. For all of the experiments, the gap height was set to 0.75 mm and the amplitude to 5.0%, which was determined as suitable by an amplitude sweep. The measurements were further conducted using the controlled deformation AutoStrain mode, in which the deflection is adjusted to a given amplitude range after every sine wave of deformation. Frequency sweeps were applied in the range of 10 Hz to 0.1 Hz in 10 K steps. The obtained frequency sweeps, in which the specimen was thermorheologically simple, were further employed to create a master curve by means of time temperature superposition (TTS). Consequently, every single frequency sweep is horizontally shifted into one master curve at a set reference temperature. The obtained melt viscosity flow profile was fit to a reduced Carreau–Yasuda equation (CY-equation, Equation (5)),

(5) $$\;\eta =\;\eta_{0}\cdot {\left[1+{\left(\lambda \dot{\gamma }\right)}^{a}\right]}^{\left(n-1\right)/a}\;$$

where *n* and *a* are constants, *λ* is a temperature-dependent relaxation time, and *η_0_* is the zero-shear viscosity [29,30]. The shift factors, *a_T_*, derived from TTS, were employed in the William–Landel–Ferry fit (WLF fit, Equation (6)) to characterize the temperature-dependent behavior of the blend’s melt viscosity.

(6) $$\;\log\left(a_{T}\right)=\frac{-C_{1}\;\left(T-T_{0}\right)}{C_{2}+\left(T-T_{0}\right)}\;$$

*C*_1_ and *C*_2_ are empirical constants, *T*_0_ is the reference temperature, and *T* is the desired temperature [31,32]. 

#### 2.2.5. Procedure to Generate the Model-Based Melt Viscosity

In our recent work, we proposed a correlation between *T_g_* and zero-shear viscosity *η_0_* of an amorphous solid dispersion and its use in HME simulation [20,21]. At a set reference temperature, the rheological flow profile of pure COP with its variables in the CY-fit and WLF-fit served as a starting point for the model-based viscosity calculation. The parameters *n* and *a* of the COP CY-fit and the variables in WLF-fit were considered as constants. The zero-shear viscosity, *η*_0_, was adjusted via the *T_g_* of the investigated blends, using our proposed *T_g_*-melt viscosity correlation (Equation (7)),

(7) $$\;\eta_{0}=a\cdot e^{b\cdot T_{g}}\;$$

where *a* and *b* are the empirically determined variables [20]. In the case of *a*, a temperature-dependency was obtained, but *b* remained constant over the investigated temperature range. To adjust *λ* of CY-fit as well, the ratio between *η*_0*,COP*_ and *η*_0*,blend*_ was calculated by Equation (8),

(8) $$a_{T,T_{g}}=\;\frac{\eta_{0,blend}}{\eta_{0,\;\;COP}}\;,\;\mathrm{and}\;a_{T,T_{g}}=\;\frac{\lambda_{blend}}{\lambda_{COP}}$$

and was applied to *λ_COP_* to identify *λ_blend_*.

#### 2.2.6. Computation of Extrusion Experiments by Using the Software Ludovic**^®^**

The simulation software Ludovic**^®^** V6.0.1 PharmaEdition (Sciences Computers Consultants, Saint Etienne, France) for hot-melt extrusion was employed. As a one-dimensional approach, it computes the non-isothermal flow conditions in the extrusion processes and calculates various parameters along the screw profile (e.g., global energy distributions, temperature, pressure, shear rate, residence time, etc.). At the first restrictive screw element, an instantaneous melting of the material is assumed. The computation begins at the die and proceeds backwards in an iterative way, until a final product temperature is reached. This procedure is needed, as the extruder is starve-fed with an unknown filling ratio [33,34,35]. In the case of conventional simulation, the physical properties and melt viscosity of the desired API/COP-blend were used. In the adopted simulation, the physical properties of the pure COP and model-based melt viscosity were employed instead [21]. Both of the simulation assumptions use the identical simulation algorithm, but the product related input parameter for simulation varies, leading to a reduced experimental effort in the case of the adopted simulation.

In more detail, the following product related input parameters need to be measured prior conventional simulation: heat capacity (solid and liquid state), density (solid and liquid state), thermal conductivity, glass transition temperature, and the melt viscosity of the formulation. For the thermal conductivity, an approximation of 0.18 W·(m·K)^-1^ is made for all of the simulations. To decrease the experimental effort in our adopted simulation approach, the following approximation of the product related input parameters were made: heat capacity and density were used from pure COP, and the glass transition temperature of the required API-polymer blend needs to be measured. The melt viscosity is estimated using the *T_g, blend_* and the rheological profile of COP (please see Section 2.2.5). 

At the end, for conducting the adopted HME simulation, only the glass transition temperature of the API-polymer blend of interest has to be experimentally determined.

#### 2.2.7. Hot-Melt Extrusion Experiments

**Figure 1:**
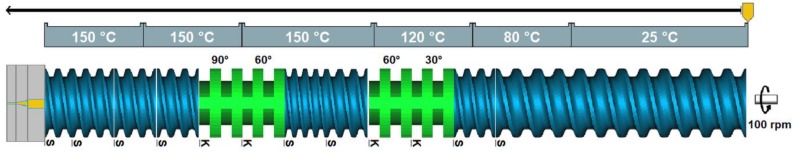
Extruder and screw configuration used with conveying elements (9, 12, and 18 mm pitch) (blue) and kneading elements (30°, 60°, and 90° staggering angle) (green). Reproduced with permission from the authors of [21], Elsevier B.V., 2017.

For performing the hot-melt extrusion experiments, a co-rotating twin-screw extruder ZE 12 (Three-Tec GmbH, Seon, Switzerland) was employed. It had a functional length of 25:1 L/D, 12 mm screws, 2 mm die, a maximum torque of 15 N·m, and a fixed screw configuration, which is depicted in Figure 1. The screw speed was set to 100 rpm and the throughput was kept constant at 2.0 g/min, using a volumetric feeding system. The specific mechanical energy (SME) during extrusion was determined by employing Equation (9),

(9) $$\;\mathrm{SME}=\frac{2\pi n\tau }{60\;m}\;$$

where *n* is the screw speed (rpm), *ṁ* characterizes the feed rate (kg/h), and *τ* is the maximum torque per shaft (N·m) with a subtracted idling speed (1.2 N·m). Every physical mixture of 60 g for HME experiments was prepared using a Turbula mixer (Willy A. Bachofen AG Maschinenfabrik, Muttenz, Swiss) for 10 min at 50 rpm. 

The amorphous state of the obtained extrudates directly after extrusion were confirmed by polarized light microscopy with and without the λ-filter (data not shown).

## 3. Results

### 3.1. API Solubility in the Polymeric Matrix and The Deviation from Couchman-Karasz Fit

**Figure 2:**
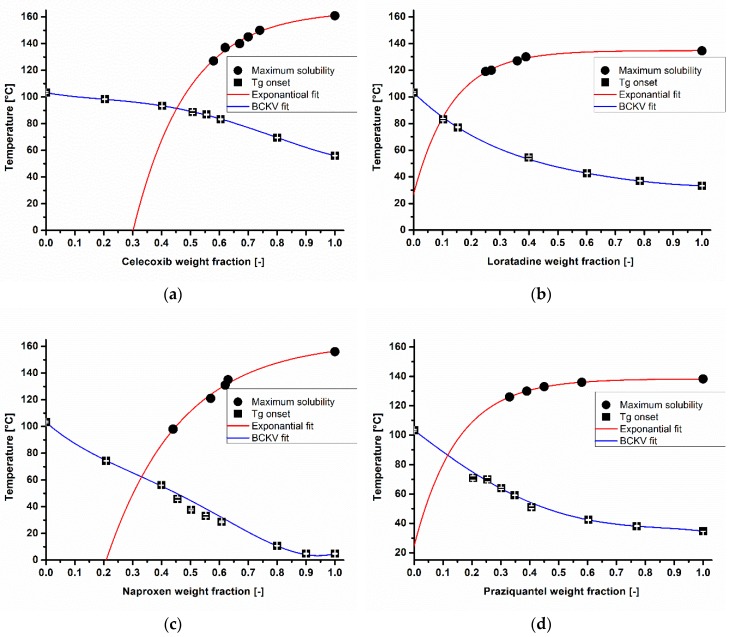
Phase diagrams of celecoxib (**a**), loratadine (**b**), naproxen (**c**), and praziquantel (**d**) in copovidone.

In general, the solubility of an API in a polymeric matrix depends on the extent of specific interactions between both materials (e.g., hydrogen bonding). To evaluate whether these specific interactions were influencing the respective melt viscosity of a mixture, phase diagrams for every API in COP were generated (Figure 2a–d). Celecoxib achieved the highest solubility of 33% in copovidone at 25 °C (Figure 2a), and a slightly lower solubility of 25% in COP was found for naproxen (Figure 2c). Regarding loratadine and praziquantel, these APIs were insoluble in copovidone at an ambient temperature and therefore the specific interactions between API and COP were assumed as negligible (Figure 2b,d). All of the conducted BCKV-fits in this publication featured a high goodness of fit (0.99 > adjusted *r*^2^). In the case of the exponential fit, a slightly lower but more than appropriate accuracy was found (0.98 ≥ adjusted *r*^2^).

**Figure 3:**
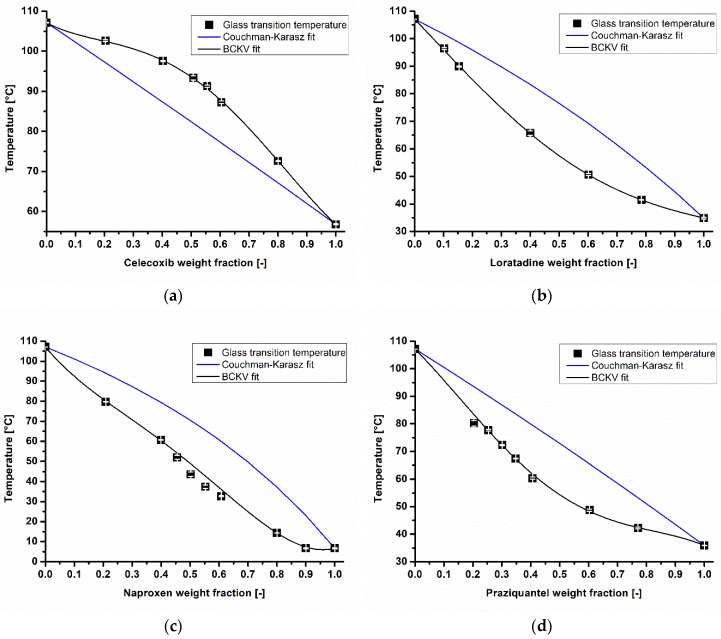
Determined glass transition temperatures (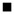), Couchman–Karasz (CK) and Brostow Chiu Kalogeras Vassilikou-Dova (BCKV) fits of celecoxib (**a**), loratadine (**b**), naproxen (**c**), and praziquantel (**d**) in different weight fractions in copovidone.

To evaluate whether a deviation from the Couchman–Karasz fit (CK-fit, Equations (2) and (3)) is influencing the accuracy of the model-based melt viscosity calculation, all of the CK-fits in comparison to the BCKV-fits (Equation (1)), and the measured *T_g_*s of the API/COP blends in different weight fractions, are shown in Figure 3a–d. Celecoxib was the only API that had a positive deviation of up to 8 °C from the CK-fit. For naproxen, a negative deviation of approximately 16 °C was obtained. A similar negative discrepancy between the CK-fit and BCKV fit of 13 and 14 °C was found for loratadine and praziquantel in COP, respectively. In general, it is reported that a deviation from the CK-fit is connected to the solubility of the API in the polymeric matrix [36]. If no deviation from the CK-fit is found, the API is assumed to be insoluble. As all of the investigated APIs deviated from the CK-fit, this connection between the deviations of the measured *T_g_*s to the CK fit and the solubility of the API in the polymer was not observed.

### 3.2. Evaluation of Potential Physical Property Changes

To investigate the influence of API and its weight fraction on the physical properties of the respective API/COP blend, the true density (*ρ*) and specific heat capacity (*c_p_*) of the physical mixtures and extrudates were measured, listed in Table 2. In the case of pure COP, the true density increased slightly from powder (1178 kg/m^3^) to the extruded material (1.191 kg/m^3^) [21]. In comparison to the physical mixtures of the COP-blends, the true density was similar to pure COP with a negligible increase of up to 4% (*ρ* ≤ 1230 kg/m^3^). Furthermore, the extrudates of all of the COP blends showed an identical increase of up to 4% in true density (*ρ* ≤ 1237 kg/m^3^). 

In the case of the specific heat capacity, a temperature-dependent increase for the pure COP from 1013 J/(g·K) at 25 °C to 1720 J/(g·K) at 150 °C was observed [21]. Regarding the API/COP physical mixtures, an increase in the specific heat capacity of up to 14% (*c_p_* ≤ 1.182 J/(g·K)) at 25 °C and at 150 °C (*c_p_* ≤ 2.003 J/(g·K)) was found, respectively. 

The only slight deviation of the true density and specific heat capacity of the COP-blends from the pure COP data were in good accordance to the findings in our recent work [21]. We already showed that a small deviation in both of the parameters did not affect the later use of the pure COP data instead of the COP-blends data, as required by the input parameters for the Ludovic^®^ simulation software. Furthermore, the accuracy of the computation outcomes for the 12 mm twin-screw extruder used was not reduced. Therefore, the small differences in the density and specific heat capacity of the ASDs compared with the pure polymeric matrix were assumed as negligible. In the case of the model-based simulation, the data of the pure COP instead of the COP-blend values were further investigated.

### 3.3. Comparison of SAOS Measurements and Model-Based Melt Viscosity Calculation

**Figure 4:**
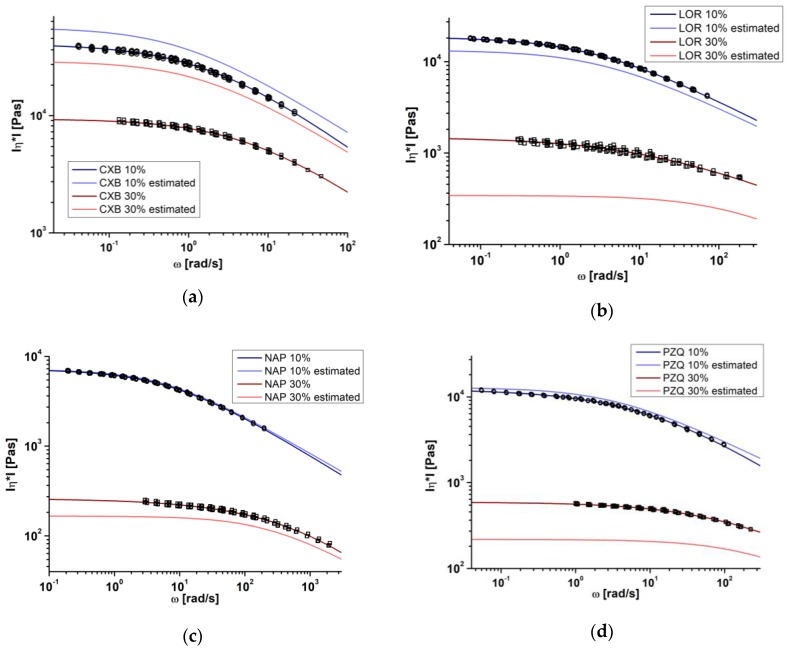
Comparison of estimated melt viscosity and small amplitude oscillatory shear (SAOS) experiments at 150 °C for celecoxib (**a**), loratadine (**b**), naproxen (**c**), and praziquantel (**d**) in copovidone.

The measured melt viscosity and the model-based melt viscosity as a function of the angular frequency at 150 °C is shown in Figure 4a–d. In general, the model-based melt viscosity of the 10% API/COP-blends were in better accordance with the measured data than the respective 30% API/COP-blends. The best agreement was found for NAP 10%, for which both of the viscosity curves were superimposed (*η*_0*,measured*_ = 7198 Pa·s to *η*_0*,estimated*_ = 7141 Pa·s) (Figure 4c). Among all of the 10% API-blends, the highest deviation between the viscosities was found for CXB 10% (*η*_0*,measured*_ = 40,791 Pa·s to *η*_0*,estimated*_ = 56,292 Pa·s) (Figure 4a). In the case of the 30% API/COP-blends, the discrepancy between the measured and model-based melt viscosity increased for both CXB 30% (*η*_0,*measured*_ = 9381 Pa·s to *η*_0*,estimated*_ = 29,114 Pa·s) and LOR 30% (*η*_0*,measured*_ = 1488 Pa·s to *η*_0*,estimated*_ = 343 Pa·s) (Figure 4a,b). However, NAP 30% showed a deviation between the model-based melt viscosity to the measured data (*η*_0*,measured*_ = 259 Pa·s to *η*_0*,estimated*_ = 165 Pa·s). In conclusion, the accuracy of the melt viscosity estimation via the blend’s *T_g_* was a function of the API weight fraction. The border for estimating the melt viscosity with a sufficient accuracy seemed to be between a 10–30% API weight fraction, dependent on the API solubility. The APIs soluble at 25 °C within COP (e.g., celecoxib) might be underestimated, whereas the insoluble APIs (e.g., loratadine) might be overestimated.

### 3.4. Energy Consumption in HME Experiments, Conventional Simulation, and Simulation Using Model-Based Melt Viscosity

**Figure 5:**
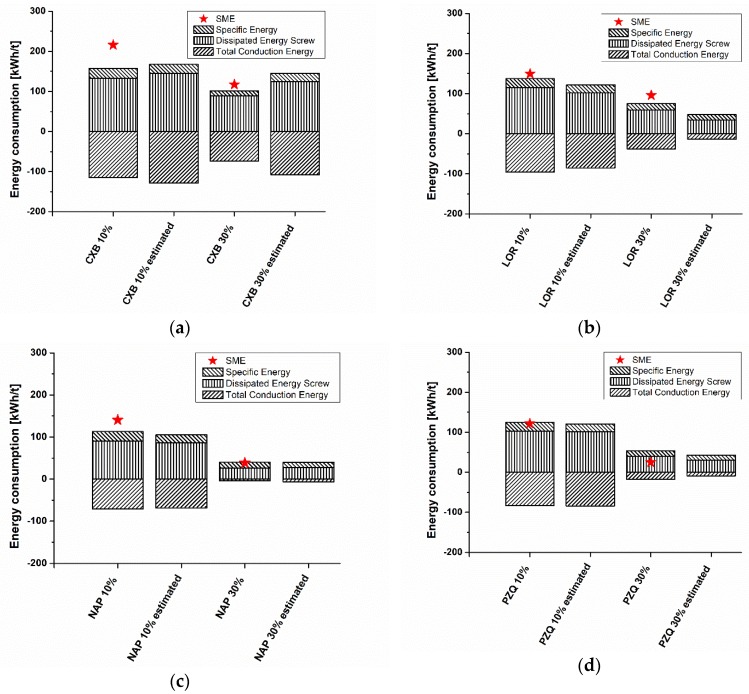
Energy consumption in hot-melt extrusion (HME) experiments, conventional simulation, and simulation using model-based melt viscosity at 150 °C extrusion temperature for celecoxib (**a**), loratadine (**b**), naproxen (**c**), and praziquantel (**d**) in copovidone.

The specific mechanical energy (SME) determined from the HME experiments was compared to the energy consumptions obtained from the conventional simulation and the simplified simulation aided by using model-based melt viscosity at an extrusion temperature of 150 °C (Figure 5a–d). In the HME simulation, SME is the sum of the specific energy, dissipated energy, and melting energy [37]. In our case, the melting energy remained unconsidered in the simulation, because the Ludovic^®^ model does not include a melting/softening temperature input for the API heat of fusion. Instead, in the relevant input parameter in Ludovic^®^, the melting temperature was filled with the glass transition temperature of the API/COP-blend. 

When all of the results are considered, because of the dependence of the dissipated energy on the melt viscosity, the dissipated energy decreased with both the lower viscosity and higher API content. The conduction energy, which determined the energy generated by barrel regulation-induced heat flux, also decreased in a similar manner. Because of the plasticizing effect of the APIs and the respective decreased melt viscosity and glass transition temperature, all of the 10% API/COP blends achieved a higher SME than the corresponding 30% API/COP blend.

In the conventional simulation and HME experiments, similar SME results were obtained. Because of the 12 mm twin-screw extruder and the volumetric feeding system that were used, a variation in the feed rate might cause the slightly higher SME to be detected in the extrusion trials compared with the computed SMEs. For highly viscous melts, a loss of energy between the gearbox of the extruder and the screws or further friction was likely, which further led to discrepancies between the measured and simulated values. Furthermore, the determined torque for calculating the SME was measured by energy power and might have uncertainties for our small-scale extruder. In the case of the 10% API/COP-blends, the highest deviation between the measured and conventional simulated SMEs were seen for CXB 10% (58 kWh/t) and NAP 10% (27 kWh/t) (Figure 5a,c). The SMEs of LOR 10% and PZQ 10% nearly superimposed instead (Figure 5b,d). A similar variance in the data between the measured and conventional simulated SMEs was found for the 30% API/COP-blends. The SMEs of NAP 30% were superimposed, whereas the highest deviation in data was found for PZQ 30% (29 kWh/t) (Figure 5c,d).

In the case of the model-based simulation, the computation outcomes were in good accordance with the conventional simulation (<20% deviation), except CXB 30% and LOR 30%. In the case of the loratadine containing samples, a higher discrepancy between the model-based and conventional SME was found. As a result of the high API-content, the model-based computation for LOR 30% differed from the conventional one and failed to calculate the correct SME. The same findings were observed for the celecoxib-containing blends. The SME of CXB 10% was simulated similarly by both approaches, but in terms of CXB 30%, the model-based simulation failed. However, the SMEs of the naproxen- and praziquantel-containing blends deviated not more than approximately 10 kWh/t from the results of the conventional simulation. For PZQ 30%, the model-based simulation fits better than the conventional one to the HME experiments, which might be coincidental. Therefore, the use of the model-based melt viscosity in the simulation led to a similar outcome as a conventional HME simulation, as long as a critical API weight fraction is not reached (approximately ≥30%) in the desired amorphous solid dispersion. Otherwise, the probability of an inadequate SME computation might increase.

## 4. Discussion

In the case of the solubility in copovidone of the four investigated APIs, celecoxib and naproxen were soluble, whereas loratadine and praziquantel were insoluble at room temperature. Regarding the deviations from the Couchman–Karasz model, celecoxib in copovidone was the only API that achieved a positive deviation from the CK-fit. For loratadine, naproxen, and praziquantel, a negative deviation from the CK-fit was observed. The assumption that a deviation from the Couchman–Karasz fit is connected to the solubility of the API in the polymeric matrix was not confirmed by our solubility findings. All of the APIs had a variation from the CK-fit, but only naproxen and celecoxib were actually soluble in copovidone. Especially regarding the 10% API/COP blends, the influence of the deviation from the CK model to our adopted HME simulation using model-based melt viscosity, cannot be confirmed. In the case of the 30% API/COP blends, only celecoxib might show a decreased simulation accuracy, related to the positive deviation from the CK model. However, the failed CXB 30% adopted simulation seems more connected to the high solubility of celecoxib in COP than to the deviation from the CK-fit.

Regarding the estimation of the rheological flow profiles of the investigated API/COP blends and the computed energy consumptions in the simulation thereof, the SMEs of the 10% API/COP blends corresponded to the experimental findings and to the conventional HME simulation. Accordingly, a 10% API weight fraction did not influence the accuracy of the resulted HME simulation, using the mainly estimated product input parameter. It leads to the conclusion that a formulation with a 10% API weight fraction can be generally be predicted by our adopted HME simulation, independent of the API’s nature. 

In the case of the 30% API content, the melt viscosity calculation and HME simulation for naproxen and praziquantel were consistent with the experimental data. The simulation of SME for celecoxib and loratadine failed, which was a result of the inadequate calculated model-based melt viscosities. The rank order from the overestimated to underestimated melt viscosity data compared to experimental results is CXB ‣ NAP ‣ PZQ ‣ LOR. This order corresponds perfectly to the solubility order in COP of CXB (33%, 25 °C) ‣ NAP (25%, 25 °C) ‣ PZQ (insoluble at 25 °C) ‣ LOR (insoluble at 27 °C). Therefore, at high API weight fractions, the solubility and the respective specific interactions between the API and the polymeric matrix might become prominent and influence the viscosity by decreasing it. In our recent work, the influence of specific interactions on the melt viscosity was already found for indomethacin, which is highly soluble in copovidone (approximately 36% at ambient temperature) [21,23]. Regarding the model-based melt viscosity, it seems that not only the glass transition temperature is important for such calculation, but also the amount of specific interactions (e.g., solubility) should be considered. If an API is less soluble or insoluble in the polymeric matrix of the formulation, our adopted HME simulation is applicable. For highly soluble APIs at high API weight fractions, the uncertainty of the HME simulation using the model-based melt viscosity is increasing and should be carefully regarded. The adopted simulation approach might fail because of the influence of specific interaction between the API and polymer, reducing the melt viscosity to an unpredictable extent.

Furthermore, the original data set for our *T_g_*-melt viscosity correlation was mainly comprising soluble APIs in the range of naproxen and less insoluble APIs. Therefore, our original data set might have triggered this uncertainty in the model-based melt viscosity calculation independent of the API solubility in COP. An improvement of the melt viscosity calculation would be the consideration of the influence of API solubility on the melt viscosity. This is still ongoing work, as the presented data set is not appropriate to allow for this consideration. Additionally, the overestimation of the CXB 30% melt viscosity might be further triggered by the rarely occurred positive deviation from CK-fit.

In general, the use of model-based melt viscosity for amorphous solid dispersions in the hot-melt extrusion simulation is feasible to compute energy consumptions thereof, using the Ludovic^®^ software. The experimental short-cut in the HME simulation is functional; however, the assumptions have to be improved to enable the calculation at higher API weight fractions. In accordance with our purpose to simplify the use of HME computation in early stage development and to reduce the effort needed, we were able to prove the good suitability of our model. Therefore, this procedure would enable an enhanced and fast estimate of a good starting point for extrusion trials in early drug development for amorphous solid dispersions.

## 5. Conclusions

A hot-melt extrusion numerical simulation using a model-based melt viscosity and further physical properties of the pure polymeric matrix led to accurate computation outcomes, as did the conventional simulation, with only few exceptions. All of the HME simulations, conducted using the commercial 1D simulation software Ludovic^®^, were confirmed by the energy consumptions of the HME experiments. It was shown that even APIs, which were not considered to establish the *T_g_*-melt viscosity correlation for amorphous solid dispersions in the first place, were impeccably simulated. However, the formulations with a high API weight fraction (≥30% *w*/*w*) should be regarded cautiously, as a decrease in computation accuracy is likely. This decrease in accuracy was likely triggered by the influence of specific interactions as a result of the solubility of the APIs, which were not considered in establishing the *T_g_*-melt viscosity correlation. In conclusion, reducing the experimental effort prior to the HME simulation using a model-based melt viscosity for simulation purposes, was proven as a valuable API-independent method to simplify the HME process optimization in the early formulation development for forming amorphous solid dispersions.

## Figures and Tables

**Table 1:** Physicochemical properties of the substances used. The molecular weight was taken from the PubChem Substance and Compound databases [26], all of the other parameters were experimentally determined.

Substance	Molecular Weight (g/mol)	Melting Point (°C)	Glass Transition Temperature (°C)	Heat Capacity Step at *T_g_* (J/g·K)
Celecoxib (CXB)	381.4	160.9	56.8	0.39
Loratadine (LOR)	382.9	134.6	34.9	0.30
Naproxen (NAP)	230.3	156.1	6.7 ^1^	0.23
Praziquantel (PZQ)	312.4	138.3	35.9	0.37
Copovidone (COP)	45,000–70,000	-	107	0.40

^1^ measured with 10% COP weight fraction because of the active pharmaceutical ingredients (APIs) recrystallization tendency.

**Table 2:** Glass transition temperature identified by Brostow Chiu Kalogeras Vassilikou-Dova (BCKV) fit, true density, and heat capacity of the COP blends investigated.

Mixture	*T_g_* (°C)	*ρ* (kg/m^3^) Powder	*ρ* (kg/m^3^) Extrudate	*C_p_* (J/(g·K)) at 25 °C	*C_p_* (J/(g·K)) at 150 °C
COP	107	1178	1191	1.013	1.720
CXB 10%	104	1230	1208	1.142	1.819
CXB 30%	101	1279	1237	1.011	1.878
LOR 10%	96	1215	1202	1.143	2.003
LOR 30%	75	1200	1204	1.097	1.864
NAP 10%	92	1190	1202	1.182	1.963
NAP 30%	71	1217	1209	1.117	1.883
PZQ 10%	96	1220	1200	1.165	1.899
PZQ 30%	72	1200	1209	1.104	1.862
